# Making decisions at the end of life when caring for a person with dementia: a literature review to explore the potential use of heuristics in difficult decision-making

**DOI:** 10.1136/bmjopen-2015-010416

**Published:** 2016-07-19

**Authors:** R Mathew, N Davies, J Manthorpe, S Iliffe

**Affiliations:** 1 Research Department of Primary Care & Population Health, University College London, UK; 2 Social Care Workforce Research Unit, King's College London, London, UK

**Keywords:** PALLIATIVE CARE, Decision-making

## Abstract

**Objective:**

Decision-making, when providing care and treatment for a person with dementia at the end of life, can be complex and challenging. There is a lack of guidance available to support practitioners and family carers, and even those experienced in end of life dementia care report a lack of confidence in decision-making. It is thought that the use of heuristics (rules of thumb) may aid decision-making. The aim of this study is to identify whether heuristics are used in end of life dementia care, and if so, to identify the context in which they are being used.

**Design:**

A narrative literature review was conducted taking a systematic approach to the search strategy, using the Centre for Reviews and Dissemination guidelines. Rapid appraisal methodology was used in order to source specific and relevant literature regarding the use of heuristics in end of life dementia care.

**Data sources:**

A search using terms related to dementia, palliative care and decision-making was conducted across 4 English language electronic databases (MEDLINE, EMBASE, PsycINFO and CINAHL) in 2015.

**Results:**

The search identified 12 papers that contained an algorithm, guideline, decision tool or set of principles that we considered compatible with heuristic decision-making. The papers addressed swallowing and feeding difficulties, the treatment of pneumonia, management of pain and agitation, rationalising medication, ending life-sustaining treatment, and ensuring a good death.

**Conclusions:**

The use of heuristics in palliative or end of life dementia care is not described in the research literature. However, this review identified important decision-making principles, which are largely a reflection of expert opinion. These principles may have the potential to be developed into simple heuristics that could be used in practice.

**Strengths and limitations of this study**

- Heuristics differ from guidelines and their use in medicine is controversial.
- This is the first review to explore the use of heuristics in dementia end of life care.
- The rapid appraisal methodology used for this search process has sourced specific and relevant literature regarding end of life dementia care.
- The remit of the search was highly focused, and the review may therefore lack the breadth of a more systematic search process. Specifically, it does not include a search of the grey literature.
- The process of determining what reasoning is consistent with heuristic decision-making is subjective; however, there was consensus between the authors regarding this.

## Background

The median survival period following a diagnosis of dementia is three and a half years.^1^ End of life care is therefore rapidly becoming one of the major priorities for practitioners in this field. The need for end of life care is growing as the population ages but the evidence base to guide those providing such care to individuals who do not have cancer is thin.^2^

Even practitioners experienced in palliative care and dementia care report a lack of confidence in meeting the needs of someone with dementia nearing the end of life.^3^ Although an European consensus is emerging for dementia,^4^ in England, the National Institute for Health and Care Excellence (NICE) guidance on end of life care is mainly targeted at patients with cancer.^5^ Recently, new guidance has been released,^6^ but this only focuses on care at the very end of life and does not encompass the needs of people with dementia. Previously, the Liverpool Care Pathway in England offered some structure for practitioners to deliver end of life care. Since its withdrawal, there is felt to have been a further decline in confidence among practitioners.^7^ ^8^

Many of the difficult decisions made for people with dementia cannot be guided by robust evidence from randomised controlled trials (RCT), because they are yet to be carried out, if indeed they are possible. There are both practical and ethical constraints, with regard to conducting RCTs in this area, and these are well documented in the palliative care literature.^9^ As a result of these limitations, there is a paucity of evidence to guide decision-making; therefore, application of ‘rules of thumb’ or heuristics may have an important role in end of life dementia care.

Heuristics are simple decision strategies, which base outcomes on only a few relevant predictors.^10^ This means they differ from guidelines. The advantages of using heuristics include precision, speed, accessibility and transparency—they can surpass the accuracy of more information-greedy decision aids and they are easy to use in a care setting, especially for those working under time constraints.^10–12^ The disadvantages are that using rules of thumb in practice is subject to cognitive bias and may perpetuate erroneous judgements; heuristics are therefore controversial. They need to be made transparent and examined critically.^13^

**Figure 1:**
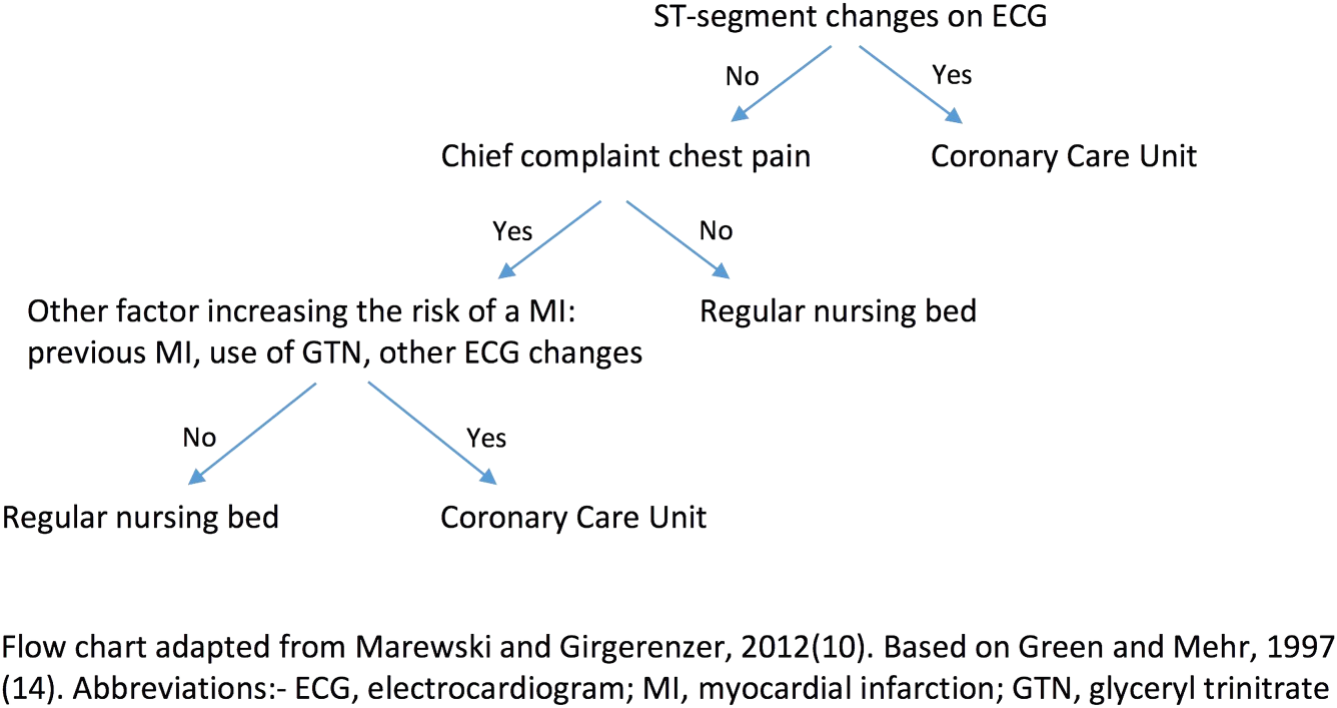
Heuristic used to determine if patients presenting to the emergency department need admission to the coronary care unit.

A commonly cited ‘fast and frugal’ heuristic is illustrated in figure 1.^10^ ^14^ Such heuristics are ‘fast’ because they do not involve much computation and ‘frugal’ because they only search for part of the information needed. As figure 1 shows, a complex decision may be simplified through the use of heuristic principles.^11^ This heuristic was developed as an alternative to a decision guide for managing patients with chest pain in some US hospitals, and deciding who needed to be admitted to a coronary care unit and who not. The Heart Disease Predictive Instrument was used to guide physicians' decisions, and contained 50 probabilities that could be checked against symptoms; it required the use of a pocket calculator. The ‘fast and frugal’ heuristic was more accurate in predicting myocardial infarctions, and had the added advantages of being both memorable and speedy.

This literature review aims to evaluate the extent to which heuristic principles are currently being used within dementia end of life care, and to identify the context in which they are being used. This literature review has been used to inform a study that will develop and test heuristics for use in dementia end of life care, through a process of co-design with family carers and health and care professionals. More information on this study and the methodology is available from Davies *et al*.^15^

## Methods

### Design

A narrative literature review was conducted taking a systematic approach to the search strategy, using the Centre for Reviews and Dissemination guidelines.^16^ Rapid appraisal methodology^17^ was used in order to keep pace with the evolution of the study and to inform the next stage of heuristic development.

### Search strategy

Four electronic databases were searched in May 2015 including MEDLINE, EMBASE, CINAHL and PSYCinfo, 1986–2015 inclusive. Reference lists and citation tracking of included papers was conducted using PubMed. A search of the grey literature was not performed.

The search used broad terms related to dementia, palliative care and decision-making.

MeSH terms and their synonyms were also used as keywords (see table 1). Online supplementary appendix 1 illustrates the search strategy used for the MEDLINE database.

**Table 1:** Database search terms

Search terms	
Dementia	Dementia, Alzheimer disease, delirium, amnestic and cognitive disorders, cognitive defect, cognitive impairment
Palliative care	Palliative care, terminal care, end of life care, terminally ill patients, death and dying
Decision-making	Decision-making, algorithms, decision support techniques, decision support systems, clinical judgment, heuristic, rules of thumb

10.1136/bmjopen-2015-010416.supp1 Supplementary appendix

### Inclusion/exclusion criteria

Papers were included if they were relevant to the process of decision-making at the end of life for someone with dementia. The process described had to be compatible with the concept of heuristic decision-making, whereby ‘rules of thumb’ are applied in order to reach an outcome. Therefore, we included papers that described stand-alone principles or ‘rules’ that could be used in decision-making, as well as papers which contained stepwise algorithms which resembled the framework of a ‘fast and frugal’ tree (figure 1). Owing to the paucity of experimental studies that have explored this topic, the papers were not formally scrutinised for quality.

Papers were excluded if they were not written in the English language and if they:

- Were exclusively about the ethical and legal principles related to decision-making;
- Focused on advance care planning;
- Explored issues related to surrogate or proxy decision-making.

Although, the heuristics that we seek to develop are intended to be in line with current legal and ethical practice, the aim of this review is to go beyond this and to look at decisions that are not always easily directed by law. They are also envisioned as a framework on which to base decisions when advance care plans and/or proxies may not be available.

### Selection procedure

**Figure 2:**
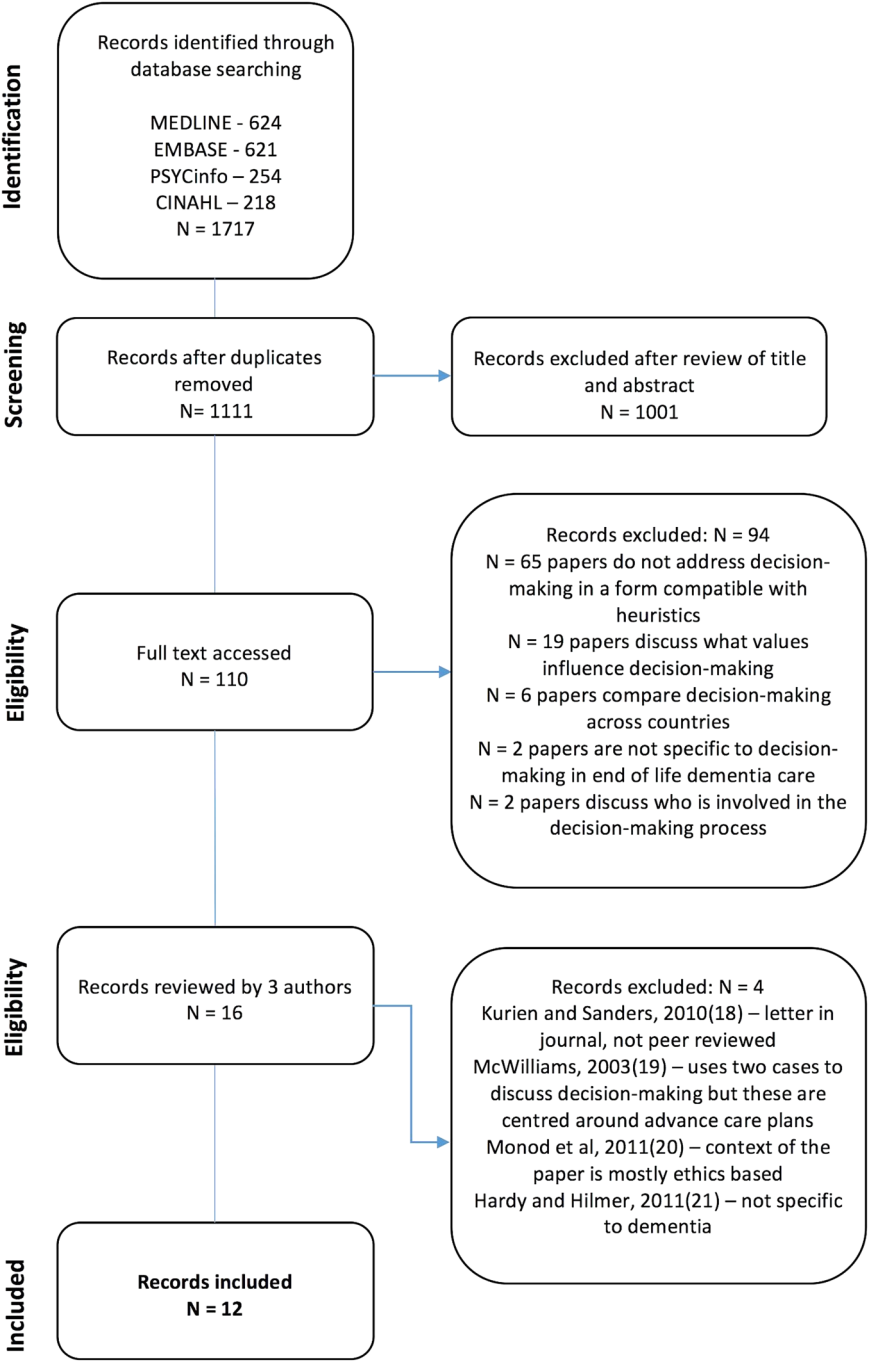
PRISMA diagram.

Titles and abstract were read and assessed for eligibility by one reviewer (RM) and the full papers of potentially relevant abstracts were retrieved and read (see figure 2^18–21^). All papers which contained some form of decision support technique/algorithm/guideline or set of principles that could potentially be used for decision-making in end of life dementia care were reviewed by three members of the research team (SI, ND and RM). Of these, 12 papers (table 2) were identified which discussed decision-making in a format which was felt to be compatible with the use of heuristics.

**Table 2:** Summary of papers included (n=12)

Author, year of publication	Country	Publication type	Management decision	Description
Cahill *et al*, 2012^22^	Republic of Ireland and Northern Ireland	Peer-reviewed paper	Ensuring a ‘good death’	Guidelines for nursing homes delivering end of life care, which have been developed from in-depth qualitative interviews with bereaved caregivers of people with dementia
Callahan, 1995^23^	USA	Report	Ending life-sustaining treatment	Report which raises the question; under what circumstances should life-sustaining treatment for someone with dementia be ended?
Gillick, 2001^24^	USA	Peer-reviewed paper	Swallowing/eating difficulties	Provides a stepwise approach about what nursing homes should do when someone with dementia stops eating
Karlawish *et al*, 1999^25^	USA	Peer-reviewed paper	Ensuring a ‘good death’	Describes how to reach consensus in decision-making, using a case study of a person with dementia who develops neurogenic dysphagia and aspiration pneumonia
Kovach *et al*, 2012^26^	USA	Peer-reviewed paper	Pain and agitation	Quasi-experimental study of a 5-step and 9-step decision support tool for management of pain and agitation in people with dementia
McAlister *et al*, 1989^27^	Canada	Peer-reviewed paper	Swallowing/eating difficulties	Proposes an analytic approach to decision-making for people with dementia who refuse feeding by hand
Palecek *et al*, 2010^28^	USA	Peer-reviewed paper	Swallowing/eating difficulties	Proposes ‘comfort feeding only’ as a means to eliminate the apparent care–no care dichotomy assumed by a decision to forgo artificial hydration and nutrition
Schwartz *et al*, 2014^29^	USA	Peer-reviewed paper	Swallowing/eating difficulties	Synthesis of literature which supports the notion that forgoing artificial nutrition and hydration is acceptable in dementia end of life care. Discusses decision-making principles when considering artificial nutrition and hydration as an option for feeding
Smith *et al*, 2009^30^	UK	Peer-reviewed paper	Swallowing/eating difficulties	Contains an algorithm to aid decisions about eating
van der Steen *et al*, 2000^31^	The Netherlands	Peer-reviewed paper	Treatment of pneumonia	Through the use of case studies, describes the use of a checklist to make decisions about whether or not to treat pneumonia in patients with dementia
van der Maaden *et al*, 2014^32^	The Netherlands	Peer-reviewed paper	Treatment of pneumonia	A 5-round Delphi study, from which a guideline was created for optimal symptom control for patients with dementia who develop pneumonia
Zagaria, 2015^33^	USA	Peer-reviewed paper	Rationalising medication	Promotes a philosophy of stopping any medication that is not in line with the primary goals of care

## Results

Although none of the papers explicitly referred to the use of heuristics, there was consensus among the authors of this present review, that the principles discussed in the 12 papers resembled the concept, and met the inclusion criteria (table 2). Findings from the publications were categorised into six main themes, as discussed below.

### Swallowing/eating difficulties

#### Establishing goals of care

McAlister *et al*^27^ developed a decision-making process which is based on establishing the goals of care. They raise the question of whether the goal of feeding is to prolong life, provide comfort or to ensure the provision of an adequate calorie intake during an intercurrent illness, in which swallow and/or appetite may be temporarily affected. Decision-makers are advised to consider the likelihood of achieving the goals of care, when considering available feeding options. This process inherently involves weighing up the risks and benefits of hand feeding versus other methods or artificial nutrition and hydration.

#### Identifying a potentially reversible cause

Attempting to identify and treat a potentially reversible cause of poor swallow and appetite appears to be a crucial part of the decision-making process. This is reflected in the approach by McAlister *et al*,^27^ Smith *et al*^30^ and Gillick,^24^ who all advise that a thorough clinical assessment should be the first step in the decision-making process. The purpose of this is to prevent diagnostic overshadowing, and ensure that practitioners identify and treat any potentially reversible causes of poor swallow and appetite, that may otherwise be overlooked. A bedside assessment of swallow by a speech and language therapist is recommended by Gillick,^24^ but Smith *et al*^30^ also advocate the use of more objective measures, such as video fluoroscopy. It is clear that identifying the cause of poor swallow and appetite is not always straightforward, as the person with dementia is often unable to express themselves or communicate their difficulties. If, however, a potentially reversible cause is thought to be contributory, a time-limited trial of nasogastric tube feeding is deemed acceptable, during which time the person's response to treatment should be closely monitored.^29^

#### Hand feeding over artificial nutrition and hydration

This literature reveals a growing consensus that hand feeding is the preferred method, when reduced eating is deemed to be a consequence of dementia and not another potentially reversible cause. Schwartz *et al*^29^ present the findings of their literature review which concludes that a decision to forgo artificial nutrition and hydration is both acceptable and in line with current scientific evidence. Of note, there is no information about the methodology of the Schwartz *et al* search or the inclusion/exclusion criteria. Nonetheless, this approach is also concurrent with the recommendations of other papers included in this present review. Gillick^24^ suggests empirically modifying food and hand feeding to whatever extent is tolerated. Smith *et al*^30^ suggest feeding techniques whereby food is offered but not forced. The use of mouth care, ice chips and artificial saliva are recommended in order to promote oral comfort. When feeding remains problematic despite these measures, it is encouraged that constant and distressing attempts to assist feeding are reviewed, and possibly discontinued.^30^ The use of artificial nutrition and hydration is actively discouraged, and instead practitioners are advised to use this time as an opportunity to discuss palliative care.^24^

#### Framing the conversation

Finally, Palecek *et al*^28^ focuses on the dialogue around feeding decisions. They suggest using the term ‘comfort feeding only’ to reframe discussions about hydration and nutrition, in an attempt to remove the apparent ‘care–no care’ dichotomy assumed by a decision to forgo artificial nutrition and hydration. They define ‘comfort feeding’ as a process which involves the skills of hand feeding, mouth care, therapeutic touch and attempting conversation with the individual with dementia while feeding. Palecek *et al* go beyond the recommendations of the other papers by highlighting the importance of terminology and phrasing, when discussing what is often an emotionally fraught subject for families and practitioners.

### Treatment of pneumonia

van der Steen *et al*^31^ evaluate the use of a checklist developed in the Netherlands to make decisions about whether or not to treat pneumonia. Health professionals are asked to consider the expected effectiveness and the potential burden of treatment. This is also in line with the approach put forward by van der Maaden *et al*,^32^ which advocates making treatment decisions by balancing life expectancy against the undesirability of life extension.

van der Maaden *et al*^32^ also specifically offer advice around symptom control in pneumonia, for people with dementia at the end of life. Their recommendations are based on a five-round Delphi study involving a panel of 24 experts. Moderate consensus was reached for 80% of the statements, of which the majority are comparable with ‘rules of thumb’. These rules are summarised below:

- Give oxygen if shortness of breath is burdensome;
- A burdensome cough warrants opioids;
- There is no evidence that anticholinergics reduce sputum retention and rattling breath;
- If opioids cause delirium, lower the dose, change route or rotate type;
- If life expectancy is 1–2 days, treatment of delirium or constipation may be unnecessary or burdensome;
- Non-pharmacological treatment of delirium is of major importance; families are likely to have an important role in this;
- In patients with chronic obstructive pulmonary disease and shortness of breath—use corticosteroids, bronchodilators and opioids.

### Pain and agitation

Kovach *et al*^26^ evaluate the use of the Serial Trial Intervention, a five-step and a nine-step decision support tool used to address the problem of underassessment and undertreatment of pain and agitation among people with dementia at the end of life. Nursing home residents (n=125) with a score of 15 or less on the Mini Mental State Examination were randomly allocated to the five-step or nine-step intervention groups. The five-step intervention addresses the physical and environmental needs of the person, and then targets symptoms with interventions. This involves providing a balance between sensory-stimulating and sensory-calming activity and ensuring meaningful human interaction on a daily basis. A trial of non-pharmacological comfort is suggested before proceeding to a trial of analgesia. Consultation with other disciplines is then advised to debate the possible use of a psychotropic drug. The nine-step tool continues with the scheduled dosing of effective treatments, stopping ineffective treatments, adding adjunctive/preventative treatments and monitoring for recurrence or development of new problems.

Both tools significantly decreased discomfort and agitation from pretest to post-test, but those who received the nine-step intervention had better outcomes than those who received the five-step intervention.

### Rationalising medication

Zagaria^33^ states that residents with advanced dementia in many US nursing homes are prescribed medications of questionable benefit. Her specific recommendations are that cholinesterase inhibitors should not be prescribed for dementia, without periodic assessment of the perceived cognitive benefits and adverse gastrointestinal effects. The overall message is that medication and treatment that are not in line with the primary goals of care should be stopped. There is, however, no guidance on the specifics of managing medical comorbidities. For example, the paper does not debate if and when it may be appropriate to stop treatment for chronic conditions such as heart failure and diabetes. These are challenging questions for many practitioners, which are not addressed in the literature.

### Ensuring a ‘good death’

Both Cahill *et al*^22^ and Karlawish *et al*^25^ take the view that a good death is not only what the patient experiences, but also what their family perceives as being ‘good’. Acknowledging this by taking a shared approach to decision-making and striving for consensus are key decision-making principles that are highlighted in both papers. It is also put forward that advocating for the patient's quality of life and dignity is central to supporting decision-making around end of life care.

Karlawish *et al*^25^ explore the process of reaching consensus in decision-making. This is illustrated through the case of a patient with dementia who has neurogenic dysphagia and aspiration pneumonia. The authors suggest identifying key decision-makers and establishing dialogue in the first instance. The aim of this is to gather information about the ‘patient journey’ to date. Clinicians are then expected to provide advice to those involved in decision-making about diagnosis and prognosis, and this should be based on current evidence and clinical experience.

Cahill *et al*^22^ used information from in-depth interviews with bereaved spouses to create guidelines to support dementia end of life care in nursing homes. The authors encourage fostering a sense of partnership between nursing home staff, residents and their families. They also promote integrated working between nursing home staff and local healthcare providers, and suggest this is essential to providing good end of life care.

### Ending life-sustaining treatment

Callahan^23^ argued that no one should live longer with advanced dementia than they would have done in the pretechnological era. He advises that in late stage dementia, there should be a shift towards stopping rather than continuing treatment. In particular, if there are signs of ‘suffering’ (verbal or non-verbal), this should be an indication to stop or at least not initiate life-sustaining treatment.

## Discussion

The papers identified in this review address six main themes—swallowing and eating difficulties, treatment of pneumonia, management of pain and agitation, rationalising medication, terminating life-sustaining treatment, and ensuring a good death. The remit of dementia end of life care is broader and some pertinent issues have not been addressed, including the management of comorbidity and addressing the psychological, social and spiritual needs of the dying person. Although there is a paucity of literature in this field, the relatively limited publication output identified from this search may also reflect the rapid appraisal methodology used. The search was centred round three terms—dementia, palliative/end of life care and decision-making. Another limitation of the search strategy is that it is not inclusive of the grey literature. It may be the case that low-evidence tools which are used in practice may not feature in peer-reviewed publications, or may not be published at all. However, this is the first review to explore the use of heuristics in dementia end of life care, and it serves as a starting point to identifying their possible usefulness within this field. Moreover, the use of rapid appraisal methods was necessary in order to keep pace with the evolution of the wider study,^15^ and has enabled the sourcing of specific and relevant literature to facilitate the process of co-design of novel heuristics.

Eight of the 12 papers identified were opinion pieces and not experimental studies; therefore, it has not been appropriate to critically appraise the methodology of included papers using a framework such as the Critical Appraisal Skills Programme (CASP) tool. For the large part, we have only been able to present and summarise the reflections of expert opinion. This reiterates the lack of robust evidence to guide decision-making in end of life dementia care. The heuristics identified in this review certainly do not contradict the existing evidence base; however, they do cover areas that are less well researched. Until more evidence emerges, the use of generally recognised principles or ‘rules of thumb’ to guide decision-making may be an alternative, and presenting them in the form of heuristics may make them more accessible.

**Figure 3:**
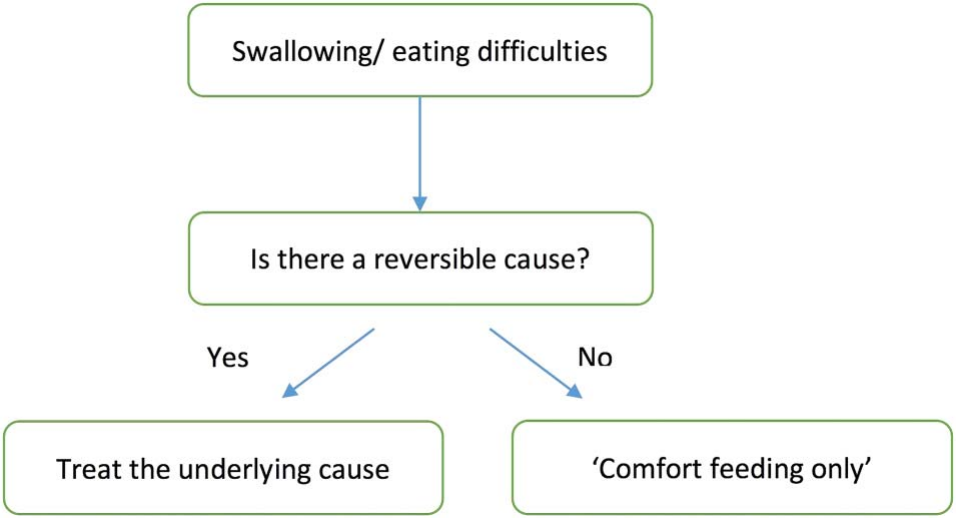
Example heuristic.

The search has identified that heuristics are not explicitly in use within the field of dementia end of life care, or at least are not reported in peer-reviewed publications. However, the decision-making principles discussed in these papers resemble heuristics, even if they are not an exact match. Unlike the decision aids described above, heuristics usually rely on the integration of information from one or two predictors to reach an end point. However, it is feasible that the concepts derived from this review could be illustrated in the form of heuristics. For example, the papers that address swallowing and eating difficulties advocate looking for a reversible cause, recognising that poor oral intake can be part of the natural progression of dementia, and using hand feeding to promote comfort and reduce distress at the end of life. The decision-making principles in these papers largely complement each other and although the points raised are varied, there are no major areas of controversy. On the basis of this search, we have developed an example heuristic addressing swallowing and eating difficulties as illustrated in figure 3.

Heuristics provide a structural framework from which intuitive thinking can be applied. They may serve to improve confidence among practitioners with regard to difficult decision-making. However, there is mixed opinion within the medical field with respect to their use. Elstein^13^ cites them as a source of many errors in clinical reasoning, but McDonald^34^ states that ‘admitting the role of heuristics confers no shame’. It is clearly an area of some controversy, and whether practitioners will embrace the use of heuristics in end of life dementia care remains uncertain.

A ‘gold standard’ in palliative dementia care is reported to be shared decision-making and person-centered care.^4^ One potential cause for concern is that the use of heuristics and decision aids may depersonalise care. When considering examples in the literature of where heuristics have been used, commonly cited illustrations are the use of heuristics to determine whether a patient with chest pain should be admitted to the coronary care unit^14^ as noted in figure 1, or whether a child with fever should be treated with antibiotics for community-acquired pneumonia.^35^ The decisions that have to be made in dementia end of life care are comparatively more complex. Being able to use heuristics while still maintaining a person-centered approach poses a potential challenge for practitioners, if these are seen as antithetical.

As previously alluded, heuristics can also be influenced by cognitive bias and can lead to poor outcomes. However, some studies confirm that heuristics can supersede both physician judgement and the use of more complex algorithms, as shown in figure 1 above.^14^ Statistical models of decision-making also demonstrate that ‘less can be more’.^11^ ^36^ Heuristics can be more accurate as predictors than complex models that use more information, a characteristic described by Gigerenzer and Gaissmeier^11^ as the ‘less is more effect’. They report a U-shaped relationship between level of accuracy and amount of information, computation or time, pointing out that there is a point where more is not better but harmful. However, the evaluation of the five-step and nine-step Serial Trial Intervention by Kovach *et al*^26^ goes against this, so whether ‘less is more’ is applicable to end of life dementia care needs further exploration.

The question of whether heuristics may be useful in end of life care for people with dementia may only be answered through a process of testing and evaluation, which will be addressed in the subsequent phases of this study. The next steps will involve the development of heuristics based on the findings of this review, and informed by interviews with family carers of people with dementia who have died,^37^ and by focus groups with carers and health and care professionals. These heuristics will then be tested and evaluated in clinical settings.^15^

## Conclusions

Although heuristics are not visible in the limited peer-reviewed literature as an explicit mechanism to aid decisions about care for people with dementia at the end of life, some simple decision rules are described which show heuristic characteristics of speed, accessibility and transparency. Such ‘rules of thumb’ could be helpful to family carers and care practitioners—who often need to make decisions under time constraints. Their utility and safety need to be examined critically.
