# Supplementary material for: Making decisions at the end of life when caring for a person with dementia: a literature review to explore the potential use of heuristics in difficult decision-making
Source: BMJ Open. 2016 Jul 19;6(7):e010416. doi: 10.1136/bmjopen-2015-010416 (PMC4964249; doi:10.1136/bmjopen-2015-010416)
Supplement: Supplementary appendix [file bmjopen-2015-010416supp_appendix.pdf]

Appendix 1: MEDLINE Search

1. exp Dementia/
2. exp Alzheimer Disease/
3. exp Delirium, Dementia, Amnestic, Cognitive Disorders/
4. dementia*.mp. [mp=title, abstract, original title, name of substance word, subject heading word, keyword heading word, protocol supplementary concept word, rare disease supplementary concept word, unique identifier]
5. alzheimer*.mp. [mp=title, abstract, original title, name of substance word, subject heading word, keyword heading word, protocol supplementary concept word, rare disease supplementary concept word, unique identifier]
6. (cognitive adj2 impairment).mp. [mp=title, abstract, original title, name of substance word, subject heading word, keyword heading word, protocol supplementary concept word, rare disease supplementary concept word, unique identifier]
7. (cognitive adj2 disorder*).mp. [mp=title, abstract, original title, name of substance word, subject heading word, keyword heading word, protocol supplementary concept word, rare disease supplementary concept word, unique identifier]
8. 1 or 2 or 3 or 4 or 5 or 6 or 7
9. exp Palliative Care/
10. exp Terminal Care/
11. (palliative adj2 care).mp. [mp=title, abstract, original title, name of substance word, subject heading word, keyword heading word, protocol supplementary concept word, rare disease supplementary concept word, unique identifier]
12. (terminal adj2 care).mp. [mp=title, abstract, original title, name of substance word, subject heading word, keyword heading word, protocol supplementary concept word, rare disease supplementary concept word, unique identifier]
13. (end of life adj2 care).mp. [mp=title, abstract, original title, name of substance word, subject heading word, keyword heading word, protocol supplementary concept word, rare disease supplementary concept word, unique identifier]
14. 9 or 10 or 11 or 12 or 13
15. exp Decision Making/
16. exp Algorithms/
17. exp Decision Support Techniques/
18. exp Decision Support Systems, Clinical/
19. decision*.mp. [mp=title, abstract, original title, name of substance word, subject heading word, keyword heading word, protocol supplementary concept word, rare disease supplementary concept word, unique identifier]
20. algorithm*.mp. [mp=title, abstract, original title, name of substance word, subject heading word, keyword heading word, protocol supplementary concept word, rare disease supplementary concept word, unique identifier]
21. heuristic*.mp. [mp=title, abstract, original title, name of substance word, subject heading word, keyword heading word, protocol supplementary concept word, rare disease supplementary concept word, unique identifier]
22. rules of thumb.mp. [mp=title, abstract, original title, name of substance word, subject heading word, keyword heading word, protocol supplementary concept word, rare disease supplementary concept word, unique identifier]
23. 15 or 16 or 17 or 18 or 19 or 20 or 21 or 22
24. 8 and 14 and 23
